# A retinal contribution to opioid-induced sleep disorders?

**DOI:** 10.3389/fnins.2022.981939

**Published:** 2022-08-05

**Authors:** Nikolas Bergum, Casey-Tyler Berezin, Jozsef Vigh

**Affiliations:** ^1^Department of Biomedical Sciences, Colorado State University, Fort Collins, CO, United States; ^2^Program in Cell and Molecular Biology, Colorado State University, Fort Collins, CO, United States

**Keywords:** opioids, addiction, sleep, circadian rhythm, melanopsin, retina, hypothalamus, suprachiasmatic nucleus

## Abstract

Chronic opioid use is linked to persistent and severe sleep/wake disturbances in patients. These opioid-related sleep problems increase risk for developing opioid dependence, mood disorders and in turn overdose in chronic pain patients receiving opioid therapy. Despite the well-established link between long-term opioid use and sleep disorders, the mechanism by which opioids perturb sleep remains unclear. Interestingly, animal studies indicate that opioids disrupt sleep/wake behaviors by altering an animal’s ability to synchronize their circadian rhythms to environmental light cycles (i.e., photoentrainment). A specific subset of retinal cells known as intrinsically photosensitive retinal ganglion cells (ipRGCs) that express μ-opioid receptors are exclusively responsible for transmitting environmental light information to sleep/circadian centers in the brain. Thus, this review will focus on the effect of opioids on ipRGCs and their projection regions that are involved in the photoentrainment of sleep/wake behaviors. Lastly, we discuss the viability of ipRGCs as a potential therapeutic target for treating opioid-related sleep/wake problems.

## Introduction

Chronic opioid use has been shown to disrupt healthy sleep/wake behavior, which often manifests as insomnia and daytime fatigue (Hartwell et al., 2014; Hallinan et al., 2019; Rosen et al., 2019; Huhn and Finan, 2021). The sleep problems associated with chronic opioid use are a significant risk factor for drug abuse, relapse and depression (Garnaat et al., 2017; Tripathi et al., 2020). Chronic pain patients are especially susceptible to the negative consequences associated with opioid-induced sleep problems, as sleep loss can exacerbate existing pain symptoms (Roehrs et al., 2006). Despite the breadth of literature documenting the negative effects of opioid drugs on sleep, the mechanism by which opioids disrupt sleep remains poorly understood. Sleep’s essential role in the successful treatment of both pain and opioid use disorders highlights the importance of developing a mechanistic understanding of how opioid drugs disrupt healthy sleep/wake behaviors.

Opioid drugs such as morphine exert much of their clinically relevant actions *via* activation of the μ-opioid receptor (MOR), with lesser contributions from interactions with the κ- (KOR) and δ-opioid receptors (DOR) (Andersen et al., 2003). These are G-protein coupled receptors that associate with inhibitory G-proteins to inhibit neuronal firing and adenylate cyclase-mediated signaling (Contet et al., 2004; Kieffer and Evans, 2009; Pasternak and Pan, 2013; Williams et al., 2013). While the MOR is credited with mediating the analgesic and hedonic properties of opioids, the KOR is implicated in the dysphoria sometimes associated with opioid use (Kieffer and Evans, 2009). DORs have also been linked to the hedonic value of opioids as well as their anxiolytic properties (Kieffer and Evans, 2009). While it is important to consider the potential contributions of all opioid receptor classes when dissecting the mechanisms that underlie chronic opioid-related sleep/wake problems, the effects of opioid drugs are primarily mediated by acting at the MOR.

Many of the sleep centers in the brain express opioid receptors and thus have the potential to contribute to opioid-induced sleep/wake changes (Eacret et al., 2020). Homeostatic as well as circadian components underlie an organism’s sleep drive (Deboer, 2018). The homeostatic drive for sleep builds the longer that the organism is awake, while circadian sleep regulation is often entrained to external factors, perhaps most notably environmental light (LeGates et al., 2014). Importantly, light has been shown to directly influence sleep onset as well as sleep homeostasis (LeGates et al., 2014).

**FIGURE 1:**
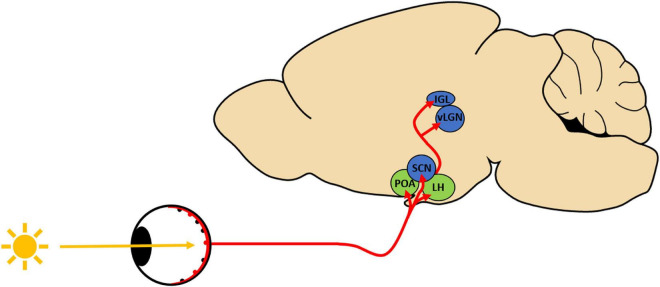
Schematic depicting intrinsically photosensitive retinal ganglion cell (ipRGC) projection regions implicated in light-induced regulation of sleep/wake behavior. ipRGCs (red) send light information *via* the retinohypothalamic tract to sleep/circadian centers in the hypothalamus (POA, Preoptic area; SCN, suprachiasmatic nucleus; LH, lateral hypothalamus) as well as to specific thalamic targets (IGL, inner geniculate leaflet; vLGN, ventrolateral geniculate nucleus). Many of these regions express opioid receptors that could underlie the opioid-induced alterations in photoentrainment. Blue: circadian photoentrainment centers; green: regions involved in light-induced sleep.

Given the importance of light in regulating sleep/wake behavior, this review assesses how opioid drugs alter the light-mediated regulation of sleep/wake behavior. Specifically, we examine the evidence that light-sensitive sleep/circadian centers within the central nervous system (CNS) contribute to the development of opioid-related sleep disturbances. In mammals, the retina is responsible for conveying environmental light into neuronal signals that can be interpreted by the brain for both image forming and non-image forming visual functions. Non-image forming visual functions, such as the light-mediated regulation of sleep/wake behavior, is dependent on intrinsically photosensitive retinal ganglion cells (ipRGCs) (Tsai et al., 2009; Schmidt et al., 2011; LeGates et al., 2014). Six subtypes of ipRGCs exist in mice (M1–M6), with each subtype exhibiting specific morphological and physiological properties as well as behavioral roles (Schmidt et al., 2011; Hu et al., 2013; LeGates et al., 2014; Li and Schmidt, 2018; Aranda and Schmidt, 2021). Similarly, five ipRGC subtypes (M1–M5) have been identified in rat and evidence suggests that M1–M4 subtypes exist in the human retina (Aranda and Schmidt, 2021). Critically, mouse studies have demonstrated that ipRGCs project to a number of brain regions that are involved in both the circadian and homeostatic regulation of sleep/wake cycles (Hattar et al., 2006; Chen et al., 2011; Schmidt et al., 2011; LeGates et al., 2014). For the purposes of this review, we will focus on M1 ipRGCs and their projection areas (Figure 1) given their functional role in the light-mediated regulation of sleep/wake behavior (Hattar et al., 2006; Schmidt et al., 2011; LeGates et al., 2014; Li and Schmidt, 2018; Aranda and Schmidt, 2021).

Many clinical studies have noted the negative impact that chronic opioid use can have on sleep/wake behavior (Huhn and Finan, 2021). Opioid receptors have been shown to be highly expressed in many regions of the brain that are involved in sleep/wake including circadian and sleep centers in the hypothalamus, as well as the locus coeruleus and the dorsal raphae nucleus (Eacret et al., 2020). Due to widespread opioid receptor expression within the brain’s complex sleep/wake circuitry, it is daunting to try and elucidate a comprehensive mechanism by which opioids disrupt sleep/wake. However, many animal studies point to opioid-induced alterations in an animal’s ability to synchronize their sleep/wake behaviors to the environmental light cycles (i.e., photoentrainment) (Cutler et al., 1999; Mistlberger and Holmes, 1999; Byku and Gannon, 2000; Meijer et al., 2000; Tierno et al., 2002; Vansteensel et al., 2003, 2005; Pačesová et al., 2015, 2016). Specifically, opioid drugs have been shown to shift the onset of circadian behavior (phase-shift), desynchronizing an animal’s sleep/wake activity with respect the environmental light cycle (Meijer et al., 2000; Vansteensel et al., 2005; Pačesová et al., 2015). While several studies attribute the opioid-induced phase-shifts to drug effects on sleep and circadian centers within the hypothalamus (Meijer et al., 2000; Vansteensel et al., 2005; Pačesová et al., 2015), the exact mechanism by which opioids can alter photoentrainment remains unclear.

In this review, we assess the evidence that opioids could alter sleep/wake behavior by acting directly on M1 ipRGCs and their projection regions involved in the light-mediated regulation of sleep/wake cycles. These projection regions can be distinguished based on their function within the context on sleep/wake regulation through parallel pathways (Zhang et al., 2021). ipRGCs photoentrain circadian centers such as the suprachiasmatic nucleus (SCN) of the hypothalamus to synchronize sleep/wake cycles to environmental light conditions (Schmidt et al., 2011; LeGates et al., 2014; Rupp et al., 2019; Aranda and Schmidt, 2021). Additionally, the acute light-induced regulation of sleep depends on ipRGC inputs to the preoptic area (POA) of the hypothalamus (Schmidt et al., 2011; LeGates et al., 2014; Rupp et al., 2019; Aranda and Schmidt, 2021; Zhang et al., 2021).

## Intrinsically photosensitive retinal ganglion cells

While there is no clinical evidence that opioids alter image forming visual functions, Grace et al. (2010) showed that pupillary light reflex (PLR) was altered in buprenorphine-treated patients. Since PLR has been shown to be ipRGC-dependent (Lucas et al., 2001, 2003; Güler et al., 2008), this suggests that opioids could alter ipRGC activity in humans. In support of this idea, M1–M3 ipRGCs have been shown to express MORs in rodents (Cleymaet et al., 2019) and MOR-selective agonist [D-Ala2, N-MePhe4, Gly-ol]-enkephalin (DAMGO) reduced ipRGC-mediated PLR in mice (Cleymaet et al., 2021). Moreover, MOR-selective antagonist D-Phe-Cys-Tyr-D-Trp-Arg-Thr-Pen-Thr-NH2 (CTAP) as well as selective ablation of MORs from ipRGCs enhanced dim light-evoked PLR (Cleymaet et al., 2021). Thus, both clinical and preclinical studies point to a potential opioid-mediated effect on ipRGC function.

Importantly, functional studies revealed that light-evoked ipRGC firing activity is suppressed by application of the MOR-selective agonist DAMGO *via* modulation of voltage-gated potassium and calcium currents (Cleymaet et al., 2019). This would suggest that opioid drugs can, in fact, modulate ipRGC activity *via* direct interactions with MORs expressed by ipRGCs. Significantly, behavioral studies also suggest that MORs expressed by ipRGCs play a role in regulating sleep/wake homeostasis *via* their interactions with the circadian oscillations in retinal β-endorphin expression (Berezin et al., 2022). These findings point to the idea that opioid-induced alteration of ipRGC activity impacts an organism’s ability to transmit environmental light information to the brain’s sleep/circadian centers.

## Circadian photoentrainment

M1 ipRGC projection areas that have been implicated in the circadian entrainment of sleep/wake behaviors to light include the SCN, as well as the inner geniculate leaflet (IGL) and the ventral lateral geniculate nucleus (vLGN) of the thalamus (Hattar et al., 2006; Chen et al., 2011; Li and Schmidt, 2018).

### Suprachiasmatic nucleus of the hypothalamus

Perhaps the region that has been most heavily implicated with the phase-shifting activity of opioid drugs is the SCN, however its exact role remains controversial (Meijer et al., 2000; Vansteensel et al., 2005; Pačesová et al., 2015, 2016). Fentanyl (a potent MOR agonist) has been shown shift the circadian phase of wheel-running activity (Meijer et al., 2000), presumably by altering light input, firing rate and circadian gene expression of hamster SCN neurons (Vansteensel et al., 2005). Interestingly, acute but not chronic morphine exposure resulted in a shift in *Per1* gene expression within the rat SCN (Pačesová et al., 2015, 2016).

Other studies have found that DOR agonists, not MOR or KOR agonists, induced a non-photic phase shift in hamsters (Byku and Gannon, 2000; Byku et al., 2000; Tierno et al., 2002). Byku et al. (2000) showed punctate DOR-labeling surrounding cells in the hamster SCN, indicative of DOR expression within presynaptic axon terminals. These studies suggest that DOR-preferring enkephalins released from the IGL onto the SCN underlie the ability of DOR-selective agonists to advance the circadian phase in hamsters (Byku and Gannon, 2000; Tierno et al., 2002).

Contrastingly, Cutler et al. (1999) reported that hamster SCN neurons were mostly unresponsive to MOR agonist morphine and enkephalins (which are known agonists of both MORs and DORs). Some studies assessing opioid receptor expression within the CNS corroborate this finding, as MORs and DORs were shown to be undetectable or lowly expressed within the mouse and rat SCN (with some evidence of KOR expression with the ventral SCN) (Mansour et al., 1994a,b; Erbs et al., 2015). While these studies offer different explanations for the SCN’s role in mediating opioid-related changes in sleep/wake rhythms, this structure is likely involved either directly or indirectly. Based on the evidence discussed here, opioids appear to alter gene expression and neuronal activity within the SCN. However, the SCN’s involvement in opioid-induced sleep/wake disturbances may not be as significant as contributions from other hypothalamic sleep centers that more robustly express opioid receptors.

### Inner geniculate leaflet of the thalamus

The IGL contains enkephalinergic interneurons as well as neuropeptide Y (NPY)-producing neurons that synapse on the SCN (Palus et al., 2017). In rat, enkephalins inhibited synaptic transmission in the IGL and robustly hyperpolarized IGL neurons (Palus et al., 2017). Critically, this opioid-induced inhibition was blocked by both MOR-and DOR-selective antagonists (Palus et al., 2017). This suggests that both enkephalinergic and NPY-ergic rat IGL neurons express functional MORs and DORs, which makes them susceptible to opioid-induced alterations in neuronal activity (Palus et al., 2017). There is also evidence of MORs in the IGL of mice and DORs in the hamster IGL (Byku et al., 2000; Erbs et al., 2015). Taken together, it is probable that the IGL at least partially mediates the phase-advances associated with systemic opioid administration.

### Ventral lateral geniculate nucleus of the thalamus

M1 ipRGCs have also been shown to the vLGN of the thalamus, another region implicated in circadian photoentrainment (Li and Schmidt, 2018; Aranda and Schmidt, 2021). While there is not much functional work regarding opioid receptor activity within this region, there is evidence suggesting the expression of MORs, but not DORs within the vLGN of the thalamus (Mansour et al., 1994b; Erbs et al., 2015). Thus, this region could also play a role in the opioid-driven alterations in sleep/wake.

## Acute light-induced sleep

Acute light-induced regulation of sleep in mice has been shown to be mediated by ipRGC inputs to the hypothalamus, specifically the preoptic area (POA) and the lateral hypothalamus (LH) (Hattar et al., 2006; Lupi et al., 2008; Muindi et al., 2014; Li and Schmidt, 2018).

### Preoptic area of the hypothalamus

The preoptic area, specifically the ventrolateral preoptic area (VLPO), has been studied extensively in regard to its role in sleep (Saper et al., 2010). In rodents, the POA contains neurons that robustly express MOR and KOR, as well as DOR to a lesser degree (Mansour et al., 1994a,b; Erbs et al., 2015). In rats, MOR and KOR mRNA is expressed in many (> 85%) of the sleep-promoting neurons in the VLPO (Greco et al., 2008). Indeed, behavioral experiments corroborate this finding, as an infusion of a MOR-and KOR-selective agonists into the VLPO increased wakefulness in rats (Greco et al., 2008). In line with this, another group found that morphine microinjections into the VLPO increased wakefulness and suppressed sleep in rats (Wang et al., 2013). This study also characterized the electrophysiological properties of VLPO neurons, specifically showing that sleep-promoting VLPO neurons were sensitive to morphine-induced inhibition of action potential firing (Wang et al., 2013). The application of MOR-and KOR-selective antagonists confirmed the contributions of both receptor subtypes in the morphine-mediated suppression of firing activity (Wang et al., 2013). These findings provide strong evidence suggesting opioid-induced inhibition of VLPO neurons may underlie the wake-promoting effects of opioid drugs in rats.

Notably, this region contains two separate populations of Gamma Aminobutyric Acid (GABA)-ergic neurons that are thought to be important mediators of sleep. Galanin-expressing GABAergic neurons in the POA are thought to be involved in the homeostatic regulation of sleep (Ma et al., 2019), however these neurons do not receive photic input from ipRGCs (Zhang et al., 2021). The GABAergic neurons in the POA that have been shown to be light responsive are mostly corticotropin-releasing hormone-expressing cells (Zhang et al., 2021). Thus, further research must be done to dissect how opioids affect the light-responsive and/or homeostatic sleep-active neurons within this brain region to improve our understanding of how (chronic) opioids alter photoentrainment circuitry.

### Lateral hypothalamus

The lateral hypothalamus (LH) has been heavily implicated in both sleep and opioid use disorders (Harris et al., 2005; Tsujino and Sakurai, 2013), making it a logical therapeutic target for substance use disorders (Matzeu and Martin-Fardon, 2020). Orexin-producing neurons in the LH project both to the ventral tegmental area (VTA) and the paraventricular nucleus of the thalamus (PVT), which are involved in reward/addiction and sleep/wake, respectively (Harris et al., 2007; Eacret et al., 2020). Moreover, many of these orexin-expressing LH neurons have been shown to express MORs in rodents (Mansour et al., 1994a,b; Georgescu et al., 2003; Peckham and Traynor, 2006; Erbs et al., 2015). Interestingly, chronic morphine exposure decreased MOR mRNA expression in the LH, while precipitated withdrawal increased MOR mRNA expression (Zhou et al., 2006). Notably, moderate levels of KOR mRNA, but only low levels of DOR mRNA were detected in the LH (Mansour et al., 1994a).

Both endogenous opioid peptides and opioid drugs can suppress excitatory inputs to LH neurons as well as directly inhibit the firing of LH orexin/hypocretin neurons (Li and van den Pol, 2008). Interestingly, chronic opioid exposure also increased the number of orexin-producing neurons in mouse, rat, as well as human subjects (Thannickal et al., 2018; James et al., 2019). Importantly, chronic morphine reduced the opioid-mediated suppression of spike frequency in LH neurons, suggesting that chronic exposure leads to cellular tolerance (Li and van den Pol, 2008). This reduction in the effects of morphine on LH neurons suggests that it is unlikely LH neurons underlie the *persistent* sleep disturbances associated with chronic opioid use.

Additionally, LH neurons receive input from light responsive GABAergic neurons in the POA (Sherin et al., 1998; Zhang et al., 2021). As cells in both of these regions robustly express opioid receptors, characterization of opioid receptor expression within light-responsive POA projections to the LH would further clarify the roles of these regions in the context of opioid-induced alterations in sleep/wake behavior.

## Discussion

**FIGURE 2:**
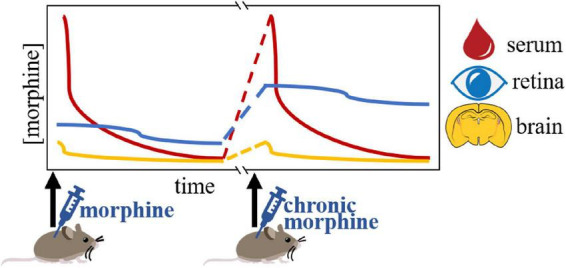
Graphical representation of the pharmacokinetic evidence for a retinal contribution to chronic opioid-induced sleep/wake disturbances (as presented in Bergum et al., 2022). Morphine levels in the retina appear to accumulate following chronic systemic exposure, while levels in both the serum and hypothalamus remain the same.

For opioid drugs to able to suppress ipRGC activity, they must deposit in the retina following systemic exposure. Previous studies have also shown that opioids deposit in the mammalian retina/VH following systemic administration (Husain et al., 2012; Gottås et al., 2016; Maskell et al., 2016; Bergum et al., 2022). Recently, our group found that morphine similarly deposits in the mouse retina following systemic injection (Bergum et al., 2022). Surprisingly, our detailed study showed that retinal morphine levels greatly exceeded the concentrations detected in the hypothalamus (Figure 2). In addition to this, we observed sustained increases in morphine levels in the retina compared to the hypothalamus following systemic administration (Bergum et al., 2022). Retinal morphine levels remained elevated up to 11 h following systemic exposure, while levels in the hypothalamus dropped to near undetectable levels just 3 h after administration (Bergum et al., 2022). This evidence suggests that morphine can persistently activate retinal targets, while morphine’s effect on hypothalamic sleep/circadian centers is restricted to just a few hours following systemic administration. Morphine’s influence on retinal MOR-expressing cells is further enhanced when we consider that chronic systemic morphine led to morphine accumulation in the mouse retina, but not the hypothalamus (Bergum et al., 2022). Notably, similar morphine accumulation was not detected in the hypothalamus, indicating that even chronic morphine only alters the activity of opioid receptor-expressing cells within hypothalamic sleep/circadian centers transiently following each systemic exposure (Figure 2). Forensic toxicological studies have shown the postmortem vitreous humor samples taken from overdose victims suggest that opioids and their metabolites enter the posterior compartment of the eye (including the retina) following systemic exposure (Wyman and Bultman, 2004). Taken together, these findings provide a strong pharmacokinetic basis for the significant contribution of MOR-expressing ipRGCs in terms of the dysregulation of photoentrainment that is associated with chronic opioid use.

This introduces a novel retinal route by which opioids could persistently alter sleep/wake behavior. This is especially exciting because retinal neurons are far more accessible for targeted interventions compared to sleep/wake centers in the brain. Targeting sleep centers in the brain to treat opioid-related sleep problems is exceedingly difficult due to the number of brain regions involved in both sleep/wake as well as other opioid-mediated effects on the body. However, the retina represents a unique CNS structure that lies outside the cranial vault, making it far more accessible for targeted therapeutic interventions.

## Author contributions

NB wrote the manuscript and created the figures. All authors edited and reviewed the manuscript for final submission.
